# Case report: Extended Isolated Stopflow Limb Infusion (EISLI) for highly malignant osteosarcoma - complete pathological tumor remission and implantation of a knee joint prosthesis

**DOI:** 10.1016/j.ijscr.2023.107918

**Published:** 2023-02-08

**Authors:** Kornelia Aigner, Emir Selak, Sabine Gailhofer, Thomas Knösel, Jayadeepa SrinivasRaju, Karl Reinhard Aigner

**Affiliations:** a Department for Tumor Biology, Medias Klinikum Burghausen, Germany; b Department for Surgical Oncology, Medias Klinikum Burghausen, Germany; c Institute of Pathology, Ludwig-Maximilians-Universität (LMU) Munich, Thalkirchner Straße 36, 80337 Munich, Germany; d Department for Oncoradiology, ATGesundheitinstitute-Rapo Yerape BH Ltd., India

**Keywords:** Isolated limb perfusion, Osteosarcoma, Extended isolated stopflow limb infusion, Regional chemotherapy, Quality of life, Chemofiltration, EISLI, Extended Isolated Stop-flow Limb Infusion, RCT, Regional Chemotherapy

## Abstract

**Introduction and importance:**

An 18-year old osteosarcoma patient with a huge tumor mass at the distal femur and inguinal metastases was treated with the intention to preserve the leg and additionally treat the pelvic metastases locally. Therefore we modulated the technique of isolated limb perfusion.

**Case presentation:**

Isolated Limb Perfusion was performed as an Extended Isolated Limb Stop-Flow Infusion (EISLI) where the pelvis was included into the perfusion bed. Balloon catheters were placed in the arterial and venous bifurcation in the pelvis. For increasing the drug concentration at the tumor site, an angiographic catheter was placed arterially with the tip right in front of the tumor region. A Stop-Flow phase before the perfusion phase was applied.

**Clinical discussion:**

After 4 cycles of EISLI the lesions in the pelvis disappeared and surgical resection of the tumor and implantation of an endoprosthesis was possible and successful. Histopathological findings showed no vital cells in the resected tumor region. Currently the patient is tumor free and does not show recurrence or pulmonal metastases for 18 months after the last induction treatment cycle.

**Conclusion:**

With EISLI the inclusion of the pelvis is possible during isolated limb perfusion. In addition with low total dosages EISLI enabled drug concentrations many times higher at the tumor site than possible during systemic chemotherapy or standard isolated limb perfusion. It is a technique that allows limb preservation and treatment of positive lymphnodes in the groin. Quality of life is maintained during the Regional Chemotherapy (RCT).

## Highlights

• Isolated Limb Perfusion can be extended to the pelvic region with balloon catheters at the arterial and venous bifurcation.
• Drug concentrations at a tumor can be increased by stopflow infusion.
• Increased drug concentrations during 5 min stopflow phase might cause sufficient tissue drug uptake.
• Extended Isolated Limb Infusion (EISLI) can be an alternative to limb amputation.

## 1 Introduction and importance

Osteosarcomas are rare, mostly highly malignant tumors which peak in frequency in the second decade of life, usually between the ages of 14 and 19. They arise predominantly in the metaphyses of long bones such as the humerus, femur and tibia and are characterized by rapid growth and early metastases particularly into the lungs.

Therapeutically, early combination chemotherapy is indicated to avoid distant metastases and to achieve resectability through tumor volume reduction [1].

Whenever possible, various surgical techniques are used to remove the tumor with free resection margins to preserve the extremity. If the tumor is not resectable with free margins due to its location and extent, amputation can often no longer be avoided. Well known methods for high- effectiveness is isolated limb perfusion and isolated limb infusion which have been standard treatments for melanoma and were developed by Thompson et al. [2], [3], [4], [5], [6]. Isolated limb infusion has also been described as a limb saving strategy for soft tissue sarcoma patients [7]. The stop-flow infusion technique was developed by Aigner et al. and enables increased drug uptake through temporarily very high drug concentrations in a milieu with a stopped blood flow in the tumor region [8], [9], [10], [11], [12]. The combination of the stop-flow technique and the isolated limb infusion including the pelvis was administered first time and is hereby described as Extended Isolated Stop-flow Limb Infusion (EISLI).

Here we report on the case of a young female patient with a highly malignant osteosarcoma at the left femur, who received EISLI and underwent a limb conserving operation and implantation of a knee joint prosthesis. The case report was planned and written in conformity with SCARE criteria [13].

## 2 Presentation of the case

### 2.1 Preliminary clinical data - diagnosis and first line treatment

**Fig. 1:**
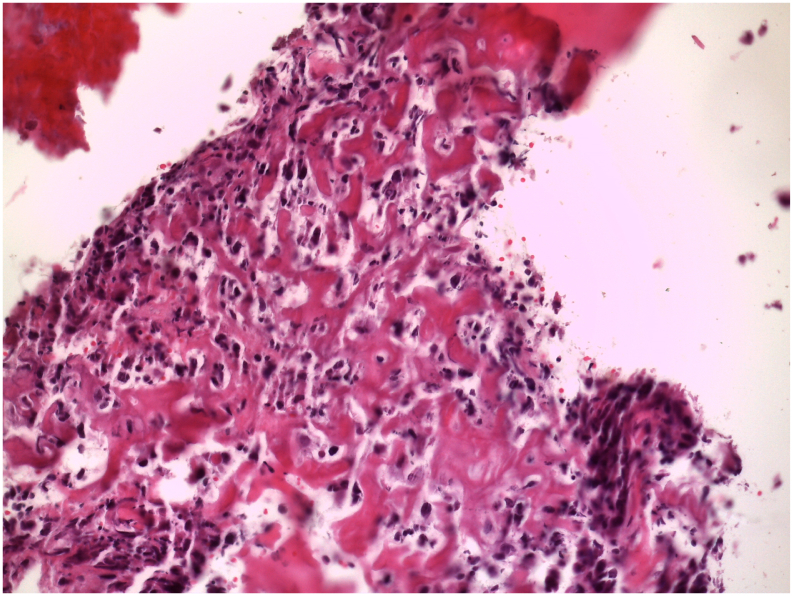
HE-staining of the initial biopsy showing a highly malignant central osteosarcoma FNCLCC grade 3.

At the time of the first diagnosis in January 2020, the patient was aged 17. After several weeks of pain in the thigh, later accompanied by swelling, an MRI showed a tumor suspicious mass in the left femur with an intra osseous craniocaudal extent of 8.2 cm, surrounded by a peri-osseous tissue growth and edema with an extent of >13 cm. The axial extent was 4.5 × 5 cm. A satellite lesion of 4 mm was located proximal to the primary mass. A biopsy revealed a highly aggressive Ostesarcoma grade G3 [Fig. 1]. She was treated in a pediatric hospital according to the EURAMOS protocol consisting of high dose methotrexate (MTX), doxorubicin, and cisplatin.

Preliminary thrombosis risk evaluations showed a homozygous methylenetetrahydrofolate-reductase (MTHFR) polymorphism (at position C667T). The activity of the affected MTHFR enzyme in homozygous carriers is known to be reduced to 30 % compared to the wildtype variant. Due to that, a reduced metabolism of Methothrexat (MTX) is present in carriers of this variant, which is shown in numerous studies that indicate increased toxicity to MTX.

Nevertheless, German guidelines do not reflect these findings and MTX was indeed part of the first line treatment for this patient from March 2020 until June 2020. Three cycles of MTX were accompanied with MTX excretion delay and severe side effects including neutropenia and exhaustive nausea. The patient decided to discontinue the treatment after four treatment cycles, although MRI response evaluation in June 2020 showed partial response and further cycles were planned.

Afterwards alternative treatments like high dose vitamin C infusions and different diets were chosen by the patient and her family during July 2020 until December 2020.

MRI and PET CT imaging in January 2021 revealed progressive disease.

The axial extent of the tumor now was 10.5 × 9 cm compared to 4.5 × 5 cm in the imaging of January 2020 with increasing metabolism, the infiltration of the bone marrow with active metabolism and a pathologic fracture of the femur. There was also a progression of the satellite tumor in the middle left thigh with increasing metabolism. The left iliac lymphnodes were increased in size and metabolic activity, thus were suspicious for metastatic relapse. Additional suspicious metastases were found in the inguinal and femoral region. No evidence of distant metastases was found.

An external orthopaedic department recommended, according to guidelines, the amputation of the leg as the only valuable and life-saving treatment option.

### 2.2 Methods - second line treatment - regional chemotherapy and surgery

However, the young lady, meanwhile 18 years, decided to refrain from an amputation in favour of regional highly concentrated chemotherapy administered by Extended Isolated Stopflow Limb Infusion (EISLI). This was performed in our facility in February 2021, with the pelvic lesions included in the isolated circuit.

**Fig. 2:**
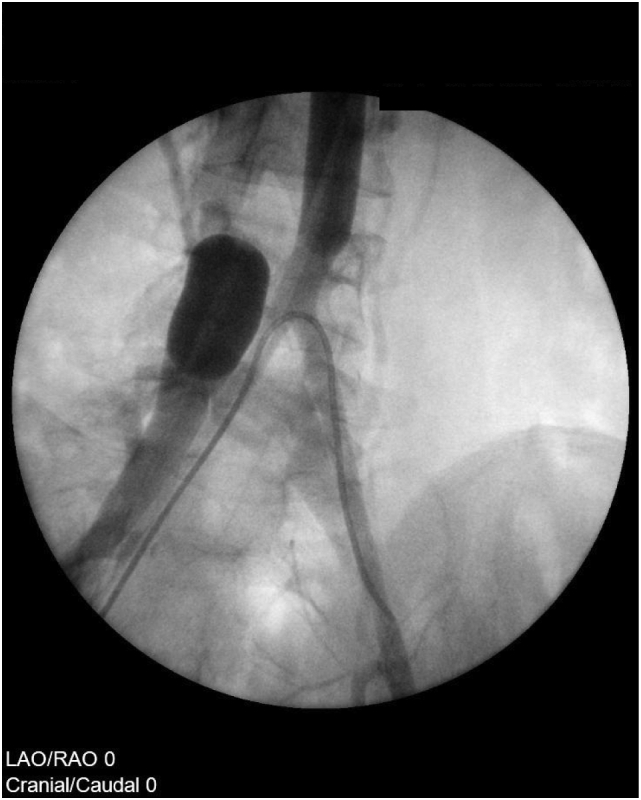
Scheme of cannulation with balloon blocking of vena cava (left balloon) and aorta (right balloon). The aortic balloon is inflated after pulsatile injection of chemotherapeutics through the angiographic sidewinder catheter in the iliac artery.

For extended isolation infusion including the pelvis with maximum cytostatic exposure of the tumor initially balloon stop-flow catheters are placed via the contralateral femoral vessels above the bifurcation of the aorta and vena cava. A Sidewinder angiocatheter is introduced into the right femoral artery proximal to the insertion site of the Stopflow balloon catheter and the tip is localized in the left common iliac artery [Fig. 2]. A pneumatic cuff is placed on the lower leg distal to the tumor.

To apply the cytostatics, the vena caval balloon is first inflated at the level of the venous bifurcation. The aortic balloon catheter remains deflated while the chemotherapy is injected in a pulsatile manner over a minute through the angiocatheter. Thereafter the aortic stop flow catheter is immediately inflated. The so-called “stop flow phase” lasts 5 min during which the tumor region is exposed to a very high concentration. The balloon catheters are then pushed up just above the bifurcations so that the entire pelvis as well as the tumor region on the knee is isolated and perfused for the next 10 min. Five minutes before the balloon catheters and the pneumatic cuff on the lower leg are deflated, chemofiltration is already underway.

The intervention was performed four times at three-week intervals. In the first cycle, 40 mg doxorubicin and 10 mg melphalan were infused as total doses. In cycles 2–4, the drug regimens were cisplatin 60 mg and doxorubicin 30 mg, respectively.

After 4 cycles of EISLI, in June 2021 the surgical excision of the tumor and implantation of a knee-joint endoprosthesis was conducted in an external orthopaedic center.

Two months later, in August 2021, an adjuvant cycle of EISLI was performed with 50 mg Cisplatin, 25 mg Doxorubicin, and 10 mg Melphalan (total dosages).

All procedures were performed in compliance with relevant laws and institutional guidelines and were approved by the institutional review committee. The work described has been carried out in accordance with The Code of Ethics of the World Medical Association (Declaration of Helsinki) for experimental treatments. Written informed consent was obtained from the patient for publication of this case report and accompanying images. A copy of the written consent is available for review by the Editor-in-Chief of this journal on request.

### 2.3 Results

All cycles of isolated stop-flow infusion were tolerated without subjective side effects or bone marrow depression. Wound healing was always undisturbed.

**Fig. 3:**
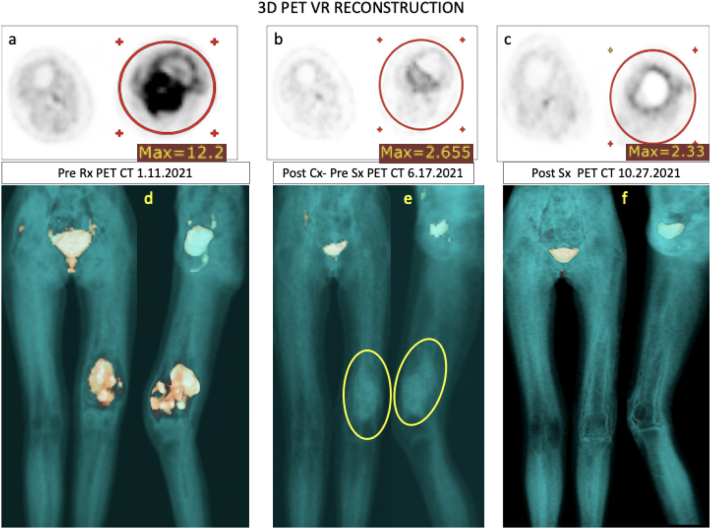
PET-CT scans: Timeline collage of lower extremity unfused PET 3D volume rendered images in anterior and lateral projections with corresponding axial unfused PET images in a & d. pretreatment appearance of an intense focal uptake in the distal left b & e. post treatment appearance of treatment responsive left distal femur with remarkable interval decrease in metabolic uptake of the primary lesion and c & f postsurgical prosthetic replacement of the diseased distal left femur extending across the knee joint devoid of pathological uptake.

An MRI after the second cycle of therapy showed tumor regression with a tumor extent of 9.8 × 6.2 × 12.2 cm compared to 10.8 × 8.9 × 13.2 cm on January 8, 2021. The edema was significantly less and the satellite nodule had shrunk from 19 mm to 16 mm and the inguinal lymph nodes were also slightly reduced in size. Clinically, the tumor appeared smaller and a complete pain response was achieved. After four EISLI cycles in June 2021, another MRI revealed further tumor regression while the satellite nodule was still present. All lymph node metastases, inguinal and iliac, had disappeared and there was no evidence of distant metastases [Fig. 3].

The diagnostic findings now allowed the surgical removal of the tumor and the implantation of a knee joint endoprosthesis.

The histopathological findings were that the entire tumor bed had been excised and showed clear regressive cell transformations with no viable tumor cells in the 12.5 cm long excision bed, as a sign of pathological complete remission. The satellite tumor was included in the excision bed without evidence of viable tumor cells. The resection margins were tumor-free.

The fifth, adjuvant cycle of EISLI in August 2021 was well tolerated with no side effects. A PET-CT in October 2021 showed neither evidence of local recurrence nor evidence of lymph node or distant metastases.

#### 2.3.1 Pharmacokinetics

**Fig. 4:**
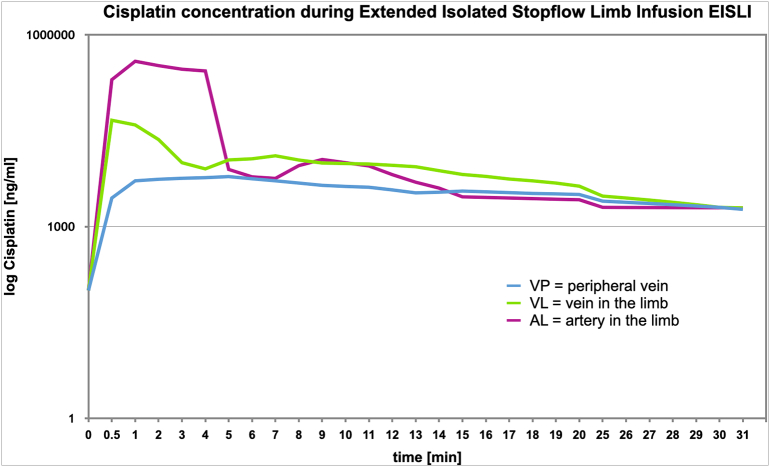
Serum concentration measurements for Cisplatin: On a logarithmic axis measurements of cisplatin concentrations are shown for the tumor afferent artery in the limb (AL), the tumor efferent vein in the limb (VL) and the peripheral vein that is outside the isolated circuit (VP). The decreasing concentration from tumor afferent artery to tumor efferent vein shows significant drug uptake into the tumor region. The concentrations in the periphery are constantly low.

The measurement of the cisplatin level during the infusion and stop-flow phase of the 4th therapy cycle showed a maximum of 386 μg/ml cisplatin in the artery supplying the tumor, with a plateau phase with high concentrations being measured during 5 min despite the low total doses. The concentration measured in the tumor's peripheral vein was still higher than usual systemic chemotherapies and reached a peak of 46 μg/ml. However, it was much lower than at the arterial measurement point, indicating stringent tissue uptake. The arterial and venous measuring points in the extremity, decreased rapidly in drug concentrations after the start of chemofiltration. The peripheral measurement showed continuously low systemic cisplatin concentrations with a maximum of 6 μg/ml [Fig. 4].

## 3 Discussion

The basic strategy for any sarcoma treatment is induction chemotherapy followed by surgical removal of the tumor and further chemotherapy. The aim of the surgical intervention is to preserve the affected extremity as far as possible, i.e. to resect the tumor with histologically tumor-free margins. Various surgical procedures are available for this, depending on the size and anatomical location of the tumor. However, a prerequisite for this is that the tumor responds well to induction chemotherapy, and the prognosis also depends on this [14].

In the present case of a highly malignant, rapidly proliferating, and very large tumor, there was some initial response to chemotherapy, but the patient refused to continue treatment after 3 cycles because of intolerable toxicity.

In principle, locally higher concentrated intra-arterial therapies came into consideration as alternatives, which had consistently good clinical approaches and good results, but no sweeping successes.

The level of effective concentration of cytostatics that can be achieved does not depend on the dose applied, but on the technique used. The administration of chemotherapeutic agents in an isolated extremity perfusion into the priming solution in the reservoir of the heart-lung machine inevitably leads to initial dilution before the chemotherapeutic agent reaches the tumor. In our present case, it is infused into the perfusion circuit as a short arterial infusion and then a so-called stop-flow phase is followed for 5 min before the isolated perfusion is started using an external roller pump. A maximum concentration of 386 μg/ml cisplatin is reached with an arterial infusion duration of 5 to 10 min of relatively low doses into the arterial circulation, whereas the tumor-toxic effect is increased by a factor of ten. In our patient, this led to complete necrosis of the osteosarcoma and affected iliac lymph nodes, allowing resection and implantation of a knee joint prosthesis. After 18 months follow up, assessments have shown no evidence of disease.

The important positive effect of EISLI was that due to concomitant chemofiltration there were never any stressful side effects and the quality of life was always maintained.

## 4 Conclusion

EISLI (Extended Isolated Stopflow Limb Infusion) is an effective treatment technique for malignant tumors of the extremities, even if metastases in the pelvic region are present. It is an extended and advanced mode of the well-established Isolated Limb Perfusion (ILP). It can lead to rapid tumor shrinkage without significant side effects and achieve resectability, even of large tumor masses, where amputation had been considered the only remaining option. Due to direct infusion into the tumor supplying artery, and the avoidance of dilution of chemotherapeutic drugs in the perfusion reservoir, extremely high drug concentrations are achieved at the tumor site despite the use of only small total dosages. The quality of life is largely preserved.

## Consent

Written informed consent was obtained from the patient for publication of this case report and accompanying images. A copy of the written consent is available for review by the Editor-in-Chief of this journal on request.

## Ethical approval

Approval for this research and publication has been given by the Medias internal review board (protocol number MIRB20221201).

## Sources of funding

This research has been financed by Medias Klinikum Burghausen, Germany.

## Author contribution

Authors contributed as follows:

Kornelia Aigner 1,2,3
Emir Selak 1,3
Sabine Gailhofer 1,3
Thomas Knösel 1,3
Jayadeepa SrinivasRaju 1,3
Karl Reinhard Aigner 1,2,3

(1) The conception and design of the study, or acquisition of data, or analysis and interpretation of data, (2) drafting the article or revising it critically for important intellectual content, (3) final approval of the version to be submitted.

## Guarantor

Karl Reinhard Aigner.

## Research registration (for case reports detailing a new surgical technique or new equipment/technology)

The technique has been registered at Research Registry and is indexed under researchregistry8553.

## Declaration of competing interest

All authors have no conflicts of interest.

## References

[1] Rümenapp C., Smida J., Gonzalez-Vasconcellos I., Baumhoer D., Malfoy B., Hadj-Hamou N.S., Sanli-Bonazzi B., Nathrath M., Atkinson M.J., Rosemann M. (2012 Sep). Secondary radiation-induced bone tumours demonstrate a high degree of genomic instability predictive of a poor prognosis. Curr. Genomics.

[2] Kroon H.M., Huismans A.M., Kam P.C., Thompson J.F. (2014 Mar). Isolated limb infusion with melphalan and actinomycin D for melanoma: a systematic review. J. Surg. Oncol..

[3] Huismans A.M., Kroon H.M., Haydu L.E., Kam P.C., Thompson J.F. (2012 Sep). Is melphalan dose adjustment according to ideal body weight useful in isolated limb infusion for melanoma?. Ann. Surg. Oncol..

[4] Thompson J.F., Lai D.T., Ingvar C., Kam P.C. (1994 Mar). Maximizing efficacy and minimizing toxicity in isolated limb perfusion for melanoma. Melanoma Res..

[5] Thompson J.F., Kam P.C., Waugh R.C., Harman C.R. (1998 Apr-May). Isolated limb infusion with cytotoxic agents: a simple alternative to isolated limb perfusion. Semin. Surg. Oncol..

[6] Thompson J.F., Kam P.C. (2004 Oct 1). Isolated limb infusion for melanoma: a simple but effective alternative to isolated limb perfusion. J. Surg. Oncol..

[7] Mullinax J.E., Kroon H.M., Thompson J.F., Nath N., Mosca P.J., Farma J.M., Bhati R., Hardmann D., Sileno S., O'Donoghue C., Perez M., Naqvi S.M., Chen Y.A., Gonzalez R.J., Zager J.S. (2017 Apr). Isolated limb infusion as a limb salvage strategy for locally advanced extremity sarcoma. J. Am. Coll. Surg..

[8] Stephens F.O. (1995 Oct). Induction (neo-adjuvant) chemotherapy: systemic and arterial delivery techniques and their clinical applications. Aust. N. Z. J. Surg..

[9] Ben-Ari G.Y. (1997 Sep). Treatment of abdominal malignancies using total abdominal stop-flow infusion and hypoxic perfusion. Gan To Kagaku Ryoho.

[10] Aigner K.R., Gailhofer S., Brammer C. (1998 Jan). Abdominal stop flow infusion breaks drug resistance in systemically pretreated progressive FIGO IIIc and IV ovarian cancer. Gan To Kagaku Ryoho.

[11] Guadagni S. (2003 Dec). Loco-regional perfusions (stop-flow techniques): state of art and future. J. Exp. Clin. Cancer Res..

[12] Aigner K., Vashist Y.K., Selak E., Gailhofer S., Aigner K.R. (2021 Nov 15). Efficacy of regional chemotherapy approach in peritoneal metastatic gastric cancer. J. Clin. Med..

[13] Agha R.A., Franchi T., Sohrabi C., Mathew G., for the SCARE Group (2020). The SCARE 2020 guideline: updating consensus Surgical Case Report (SCARE) guidelines. Int. J. Surg..

[14] Ritter J., Bielack S.S. (2010 Oct). Osteosarcoma. Ann. Oncol..

